# Indoor Microbiome and Antibiotic Resistance on Floor Surfaces: An Exploratory Study in Three Different Building Types

**DOI:** 10.3390/ijerph16214160

**Published:** 2019-10-28

**Authors:** Mridula Gupta, Seungjun Lee, Michael Bisesi, Jiyoung Lee

**Affiliations:** 1 College of public Health, Division of Environmental Health Sciences, The Ohio State University, Columbus, OH 43210, USA; gupta.285@buckeyemail.osu.edu (M.G.); lee.5178@osu.edu (S.L.); bisesi.12@osu.edu (M.B.); 2 Department of Food Science and Technology, The Ohio State University, Columbus, OH 43210, USA

**Keywords:** traffic level, floor types, carbapenem resistance, hospital, microbial source tracking, dog-specific fecal bacteria

## Abstract

Floor materials in indoor environments are known to be reservoirs of microbes. We focused on examining bacterial community composition, antibiotic resistance (AR) and microbial source tracking (MST) of fecal bacteria on the floor surfaces. Swab samples were collected from carpet and vinyl floors in three different buildings (medical, veterinary, and office buildings) from high and low traffic areas. Bacterial communities were determined with 16S rRNA sequencing, and AR (tetracycline (*tet*Q), sulfonamide, and carbapenem (KPC)) and MST (human-, canine-, avian-, and ruminant-specific fecal bacteria) were examined with quantitative polymerase chain reaction (PCR). The results show that *Proteobacteria* and *Actinobacteria* were the most abundant phyla. Traffic level significantly affected the number of operational taxonomic units. Traffic level was a key factor for distinctive bacterial community in the medical center. Targeted ARGs were detected from all buildings and *tet*Q concentration was related with traffic level, and KPC was only detected from the medical center. Most of the floor surfaces showed the presence of dog-specific fecal bacteria (83%) followed by bird-specific fecal bacteria (75%). The results suggest that traffic levels affected the bacterial levels and fecal contamination is prevalent on the floor surfaces. This is the first study that reports KPC presence on the floor surfaces.

## 1. Introduction

In the United States, humans spend up to 90% of their time in indoor environments, such as homes, offices, vehicles, and hospitals [1,2,3]. Recent studies have paid attention to spatial and temporal variations of microbial communities in indoor environments because of the concerns that microbes in indoor environments may influence some human illnesses [4,5]. Microbiological composition of an indoor habitat is influenced by many factors, including building materials, occupants, outdoor air, pets, moisture, etc. [5,6,7,8,9,10]. Previous studies have explored how microbial communities differed in different building systems (e.g., ventilation system), the process of microbial colonization in new buildings, and the microbiome’s impact on human health primarily in homes [5,6,7,8,9,10]. The study revealed that geographic location and building types heavily influence the microbial composition [11]. In the indoor conditions, many internal factors, such as surface types and materials, usage pattern, and occupants, induced differences in microbial succession [8,12,13,14,15].

The increase of microbes associated with antibiotic resistance genes (ARGs) has been an emergence issue [16]. The ubiquitous use of antibiotics on farms, antimicrobials in healthcare facilities, and overuse of antibiotics has led to increase in multi-drug resistant microbes. Numerous studies demonstrated that ARGs spread in various environments [17]. Especially, AR is prevalent in indoor environments, thus ARGs have been studied in several indoor environmental settings [18,19,20,21,22,23]. Most studies were focused on specific buildings types, such as meat-producing facilities, concentrated animal feeding operations, wastewater treatment facilities, and hospitals [18,22,23,24]. However, there are limited studies on ARGs in other common building types, such as offices. In addition, previous studies focused on airborne microbes in indoor environments.

The objective of this study was to investigate the prevalence of ARGs and bacterial compositions in the indoor environment by focusing on: (1) Two different types of floor materials—carpets and vinyl tiles; (2) different building types—human medical center, veterinary hospital, and an office building, and; (3) floor surface locations with various traffic levels. In addition, we also applied microbial source tracking tools to determine fecal bacterial sources from human and animal sources on the floor surfaces.

## 2. Materials and Methods 

### 2.1. Sampling Sites and Indoor Areas

Surface samples were collected from various sites and areas (3 building types × 2 floor types × 2 traffic types). The sampling sites and characteristics of their indoor areas are described below (Table 1):

a) Building types: Medical center, veterinary hospital and an office building which is a building having classroom, offices and laboratories. All these are buildings are located at The Ohio State University in Columbus, Ohio, USA;

b) Floor types: Carpet and vinyl tiles from each building type;

c) Traffic types: In each building, the number of visitors were counted on the sampling area to categorize as a high- or low-traffic area. Low-traffic areas were places with 15 or fewer visitors per day. High-traffic areas had 100 or more visitors per day on average.

### 2.2. Sample Collection and DNA Extraction

Samples for microbial analysis were collected using two individual sterile swabs (Copan nylon flocked swabs, Copan Diagnostics, Brescia, Italy) with 1X phosphate-buffered saline solution (Fisher Scientific, Hampton, NH, USA). A 5 cm by 5 cm area was selected for each floor area and the swab was run in zigzag fashion vertically and horizontally for ten times to cover the entire 25 cm^2^ area [25]. Total number of the swab samples was 24 (8 per each building type). The swab samples were transported to the laboratory (Ohio State University, Columbus, OH, USA) on ice. After cutting off a head of the swab, genomic DNA was extracted from the swabs using Mo Bio Power Soil DNA isolation kit (Mo Bio, Carlsbad, CA, USA). The head of the swab was placed in the bead tube of the extraction kit. All the procedures were followed according to the instructions. The final volume of elute was 100 µL. The extracted DNA was purified using Power Clean DNA Clean-Up Kit (Mo Bio, Carlsbad, CA, USA), and then the purified DNA was stored at −20 °C.

### 2.3. Detection of Antibiotic Resistance Genes and Microbial Source Tracking

Tetracycline (*tet*Q), sulfonamide (*sul*1), and Klebsiella pneumoniae carbapenemase (KPC) resistance genes were quantified [26,27,28,29]. For microbial source tracking (MST) of fecal contamination sources, human- (HF183 [30]), ruminant- (Rum2Bac [31]), dog- (BacCan [32]), and bird-specific (GFD [33]) assays were performed. All experiments were conducted in triplicate using the CFX96 TouchTM real time PCR detection system (Bio-Rad, Hercules, CA, USA).

SYBR Green PCR analysis was conducted for quantification of *tet*Q, sul1, KPC, and GFD. The total volume of qPCR mixture was 20 µL including 2 µL DNA template, 10 µL SYBR Green PCR Master Mix (Applied Biosystems, Foster City, CA, USA), and 500 nM primers. To quantify HF183, Rum2Bac, and BacCan, the TaqMan-based qPCR analysis was performed. The total volume of qPCR mixture was 20 µL including 2 µL DNA template, 10 µL TaqMan universal PCR Master Mix (Applied Biosystems, Foster City, CA, USA), 500 nM primers, and 250 nM probe. A mixture of all PCR regents with nuclease-free water (Fisher Scientific, Fair Lawn, New Jersey, USA) was used as a negative control for each PCR reaction. The PCR cycling conditions was composed of an initial cycle at 50 °C for 2 min and 95 °C for 10 min, followed by 40 cycles of denaturation at 95 °C for 15 s, annealing and extension followed by reference conditions [26,27]. After amplification, melting curve analysis for SYBR Green qPCR analysis was performed by heating samples to 95 °C for 30 s, cooling them to 62 °C for 1 min, and then heating them to 95 °C at a rate of 0.2 °C/s [34].

### 2.4. Bacterial Community Analysis

V3–V4 region of 16S rRNA was amplified with 16S universal primers for bacterial community analysis [35]. The amplification, sequencing, and basic data analysis were conducted by ChunLab Inc. (Seoul, Korea) using Miseq Platform system. Low quality sequences with less than 300 base pairs and less than 25 average quality score was filtered and discarded. ChunLab uses EzTaxon-e database (http://eztaxon-e.ezbiocloud.net/) coupled with BLASTN search tool for taxonomic classification of individual sequence each read. The similarity cutoff value for determining valid similarities between species was at 97%. The richness and diversity of samples were determined by Chao1 estimation and the Shannon diversity index at 3% distance. The bacterial composition analysis and diversity indices were calculated by CLcommunity software (version 3.46, ChunLab Inc., Seoul, Korea). The CD-HIT (Cluster Database at High Identity with Tolerance) program was used to define OTUs [36].

### 2.5. Data Analyses

The data were grouped by building types, floor types, and traffic levels for statistical analysis. The descriptive statistics were performed with STATA software (version 12.0, Stata Corporation, Texas, USA). Differences in the bacterial OTUs and α-diversity were tested using a paired t-test. Matched pair analysis was performed for traffic types by pairing with floor types in each building. Principal coordinates analysis (PCoA) was applied to visualize data similarities in β-diversity using PAST (Ver. 3.1). Permutational analysis of variance (PERMANOVA) was used to test significant differences in bacterial community structure using the PAST 3 software based on Unifrac distance matrices. Statistical significance was considered at *p* < 0.05.

## 3. Results and Discussion

### 3.1. Antibiotic Resistance

**Figure 1:**
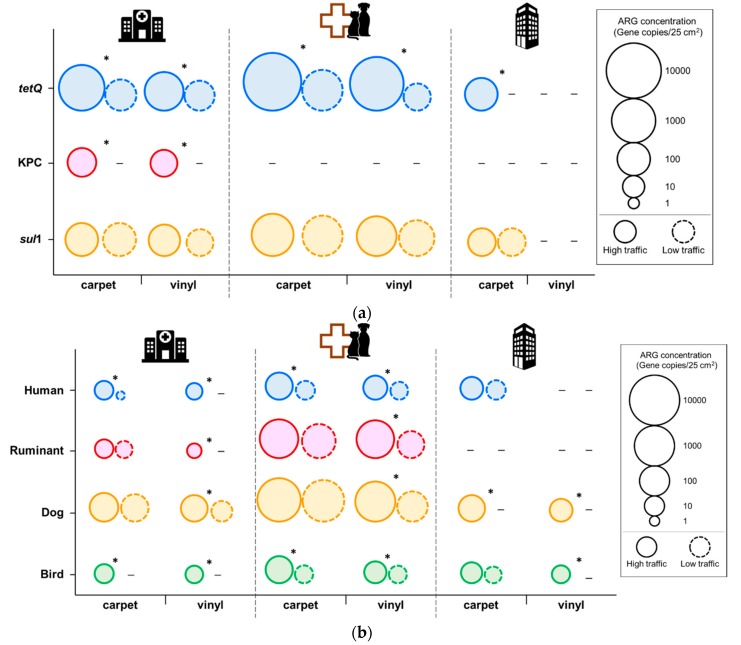
Concentrations of antibiotic resistance (tetracycline, sulfonamide, and KPC) (**a**) and fecal bacteria from human (HF183), ruminant (Rum2Bac), dog (BacCan), and bird (GFD) (**b**) in floor samples from each type of building (medical center, veterinary hospital, and office). * Statistically significant difference between the high and low traffic areas (*p* < 0.05)—Not detected.

Since humans use more than 90% of their time in indoor environments [1,2,3], it is important to examine the prevalence and concentration of ARGs in indoor surfaces. Our data demonstrated widespread distribution of *tet*Q and *sul*1 in all the sample areas, except the samples obtained from vinyl tiles of the office building (Figure 1a, Table S1). KPC was only detected from the surface of high-traffic area in the medical center, both from carpet and vinyl surfaces. The *tet*Q and *sul*1 were found to be most abundant on the carpet swab samples from the veterinary hospital. Floor samples for veterinary hospital generally had higher levels of *tet*Q and *sul*1 compared to other building types. Even though there was no significant difference between building types, traffic pattern was significantly correlated with the *tet*Q concentration in each floor type. The *tet*Q concentration was significantly higher in the high traffic area than in the low traffic area. However, the levels of *sul*1 was not significantly different between traffic patterns.

Our results imply that most indoor environments can harbor ARGs. This is a concern for possible transfer of these ARGs to other organisms, posing a potential health risk. In this study, traffic patterns showed a significant difference in the abundance of these genes; *tet*Q concentrations were significantly higher in the high-traffic areas. Previous study also demonstrated that humans are a primary vector of bacterial transmission [9]. Although building types did not differ significantly, in general, veterinary samples had higher concentrations of *tet*Q and *sul*1. Hartmann et al. showed that higher abundance of ARGs was significantly correlated with higher concentrations of antimicrobial chemicals in indoor environment [16]. ARG data show the need for further investigation to model the ARG transmission modes in different building types. It was interesting to note that KPC was only detected from the medical center floor samples. Carbapenems, which have a wide spectrum of activity of β-lactam antibiotics, are used for treatment of infections caused by multi-resistant pathogens. The spread of acquired carbapenem resistance is a critical issue for public health. Hospital floors should be considered as a potential hot spot of carbapenem resistance dissemination during the planning of AR spread intervention. To our best knowledge, this is the first study that reports KPC detection from floor surfaces.

### 3.2. Microbial Source Tracking

Interestingly, fecal contamination from human and animal sources was frequently detected. Fecal contamination was identified with MST by targeting human-, dog-, bird- and ruminant-specific fecal bacteria (Figure 1b, Table S1). Among the building types, the veterinary hospital floors, both carpet and vinyl, harbor the most fecal bacteria from all the MST markers tested. In terms of fecal contamination sources, dog-associate fecal bacteria were obviously present on most of the floor surface in all the building types (83% positive for BacCan; 5.4 × 10 to 5.6 × 10^3^ gene copies per 25 cm^2^). The highest level of BacCan was detected from the veterinary hospital. The ruminant fecal bacteria level in the veterinary hospital was also higher than other buildings. Bird- (75% positive; 2.0 × 10 to 2.5 × 10^2^ gene copies per 25 cm^2^) and human-specific fecal contamination (63% positive; 1.0 × 10 to 3.5 × 10^2^ gene copies per 25 cm^2^) was prevalent across all the building types. These results indicate that those human- and animal-originated fecal contaminations could be transmitted via human traffic, potentially by shoes. As far as we know, this is the first study to apply MST to indoor built environment floors.

### 3.3. Bacterial Community

#### 3.3.1. Bacterial Diversity and Richness

**Figure 2:**
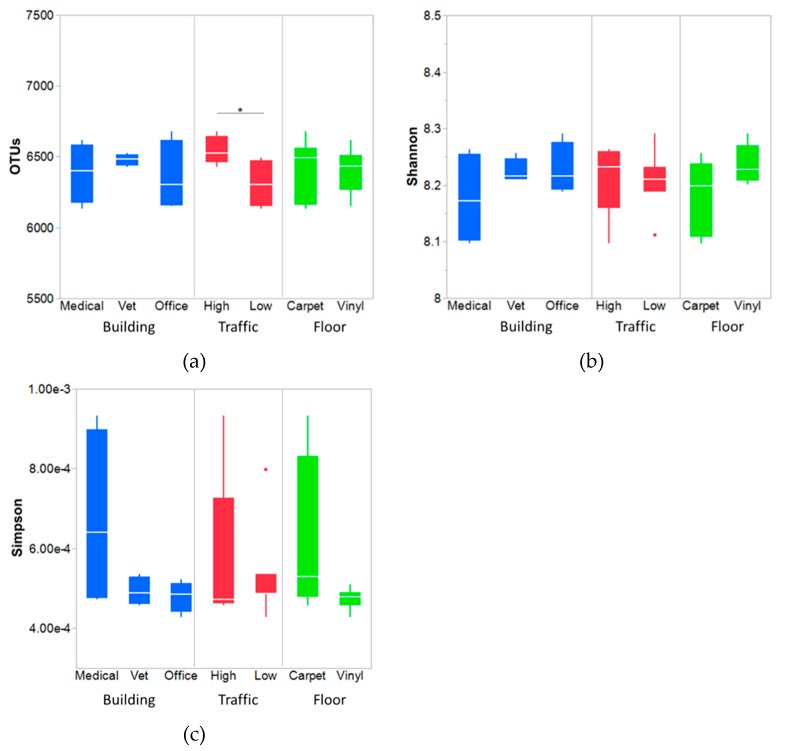
Comparison of OTUs (**a**), Shannon diversity index (**b**), and Simpson diversity index (**c**) between the floor, traffic, and building types. The box-and-whisker plot indicates the median line, 1st and 3rd quartiles. The error bars below and above of a box indicate the 10th and 90th percentiles, respectively. Outliers are shown as filled circles. * Significant difference of OTUs between the high and low traffic areas was observed (*p* < 0.05).

Figure 2 shows a summary of OTUs and diversity indices. The extremely high OTUs and α-diversity indices indicate the richness and diversity of the floor bacteria. Indoor floor surfaces have been reported to contain richer community and composition compared to ceilings and walls [10,11,12,13,14,15]. This is not surprising because of the nature of the surfaces in those buildings. Dust and soil particles from outdoors and indoors are deposited on the floor surfaces, along with bacterial cells from humans and pets [2,3,14].

Building types and floor types did not differ significantly with respect to the number of OTUs measured. There was no statistically significant difference in bacterial diversity indices between samples. However, the number of OTUs were significantly higher in high-traffic samples compared to low-traffic samples (Matched pair t-test, *p*-value = 0.018). The traffic level was matched with floor types and tested for significant difference in OTU number. Carpet samples had higher OTUs than vinyl only where traffic is high (Figure S1). It can be postulated that higher traffic brings in higher amount of outdoor soil via shoes from the visitors and occupants, along with pets, bacterial shedding as well. Hence, a higher number of microbes or bacteria is expected for those areas with a higher volume of traffic [37]. Higher OTUs, which generally mean species or individual bacterial types in higher traffic areas, implies people carry different types of bacteria. Bacterial richness was measured with Chao1 and ACE (Table S2), and the estimates from Chao1 method was at least 2–3 times higher than the observed OTUs. This indicates the presence of high number of singleton and doubleton bacterial species in the samples [38]. None of the factors, traffic, building or floor types, differed significantly in influencing bacterial richness.

#### 3.3.2. Bacterial Community Composition

**Figure 3:**
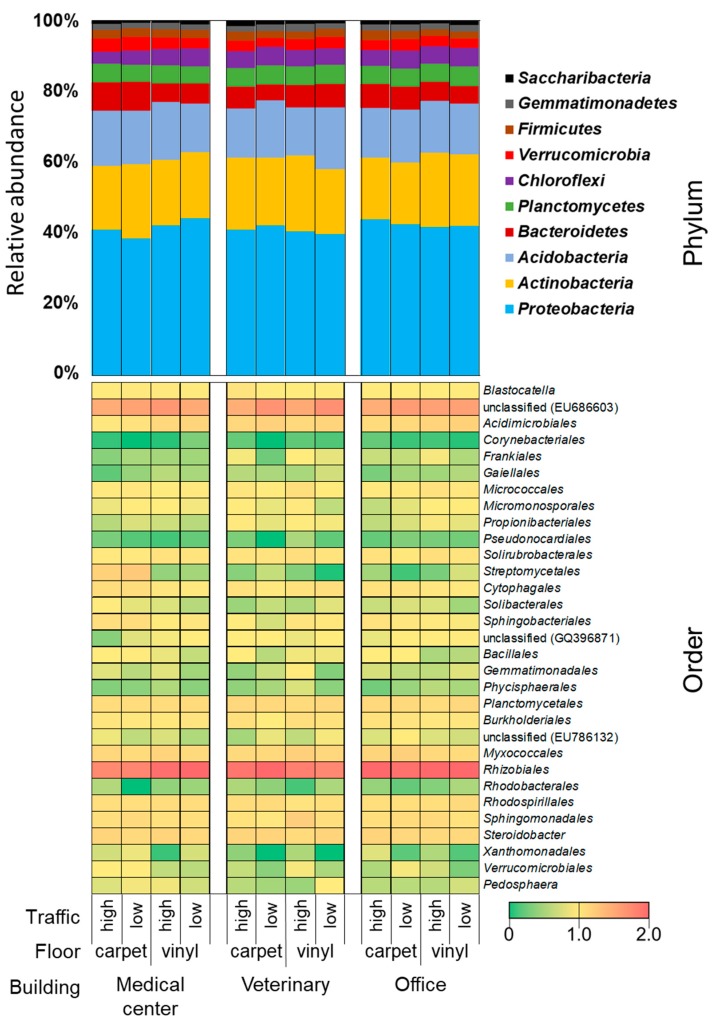
Summary of relative abundance of bacterial community (phylum and order levels) from different floor materials, traffic level and building types.

This study shows the snapshot of the microbial profile of three different building types. Major observed phyla were *Proteobacteria* (40%), *Actinobacteria* (19%), *Acidobacteria* (14%), and *Bacteriodetes* (6%) (Figure 3). The distribution of the phylum among the various building types did not differ much. The most abundant class was *α-Proteobacteria* in *Proteobacteria*, followed by *Actinobacteria* in *Actinobacteria* and an uncultured bacterium (accession no. EU686603) in *Acidobacteria* (Figure S2). Hospital floors had a higher percentage of *Actinobacteria* and *γ-Proteobacteria*. The order level bacterial composition is shown in Figure 3. *Rhizobiales* was the most abundant order in all the samples and it has been known that *Rhizobiales* are commonly found in soil. It is not surprising to see the abundance of *Rhizobiales* because all the floor surfaces might have been contacted with soils from outdoors that got deposited by foot traffic. Previous study reported that about 20–85% of indoor soils come from outdoors [39]. Lax et al. showed that bacterial community in floors was similar to the bottom of shoes [40]. Floors can also become enriched with organic and inorganic matters, and can serve as a reservoir of microbes [38]. Furthermore, previous study showed that the difference in the bacterial composition of buildings were mainly driven by moisture [41]. The bacterial composition of the samples obtained in this study was very diverse and enriched with soil bacteria, which is important since soil bacteria are known as naturally antibiotic resistant [42].

Another difference observed was that previous microbiome studies in indoor settings have found *Firmicutes* as a major phylum, but our study did not find it so [13,41,43,44]. A fair comparison cannot be made because most of the indoor microbiome studies collected samples not necessarily from floor surfaces, but rather from several other surfaces, such as door-knobs, kitchen counters and sinks [9,11,13,41,43]. Previous studies about indoor surfaces, touched directly by hands or bare skins, found *Firmicutes* as the most abundant phylum [13,41,44]. In contrast, surfaces not touched by hand directly, like floors in this study, have a bacterial composition similar to soil.

In addition, it is noteworthy to mention another group that has public health relevance. Floor samples from the hospital had a higher percentage of bacteria from *Streptomycetales* order from *Actinobacteria*. The dominance of *Streptomyces* group in the medical center can be a concern and needs further examination. *Streptomyces* are ubiquitous bacteria found in an indoor environment with soil as its source. It has been reported in vacuum dust and has also been found to be higher in moisture-damaged buildings [41,45,46]. The carpets with such a high number of *Streptomyces* in the hospital samples may be the result of frequent carpet cleaning. Hot water extraction method is commonly used for carpet cleaning in medical centers. Thus, carpet tiles can retain the moisture left after such cleaning techniques, which enable moisture-related microorganisms to survive easily.

#### 3.3.3. Bacterial Community Structure

**Figure 4:**
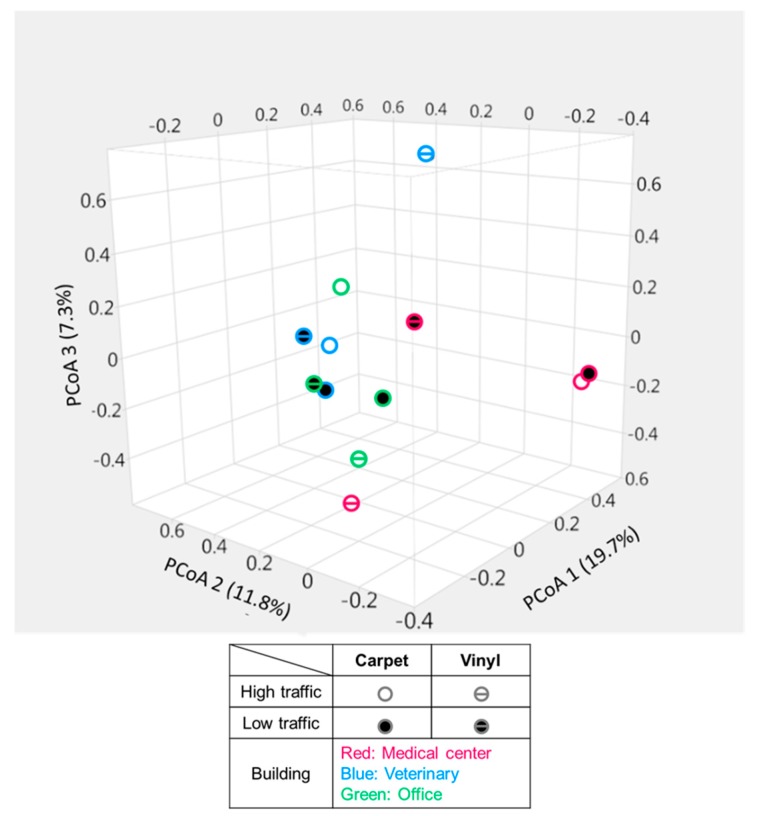
Principal coordinate analysis plot showing the similarity relationships among the bacterial community from the floor samples.

Three coordinates of the PCoA explained about 19.7%, 11.8%, and 7.3% variations in the bacterial community structure (Figure 4). UniFrac distance did not detect any meaningful clustering of samples based on building types, traffic types, or floor types. This is in agreement with previous findings that surface material did not differ in terms of bacterial composition [15]. However, both high- and low-traffic carpet samples collected from the medical center were found to be very close to each other and distant from most of the samples. PERMANOVA indicates that there are no significant groupings of the samples according to the tested categories; building, floor or traffic types (*p* = 0.05). Species diversity within the groups was bigger than between groups (e.g., buildings and traffic types), which was indicated by negative r value in Analysis of Similarity (ANOSIM) test (data not shown). Floor types had a positive, albeit small r value, indicating some difference in their bacterial composition, but it was not statistically different.

## 4. Conclusions

In summary, our exploratory study found that the floor surfaces tend to be heavily colonized by bacteria, especially soilborne bacteria. There was not much apparent difference in bacterial composition between the buildings or floor types, but traffic level plays a significant role in influencing on bacterial abundance and community. Fecal contamination was prevalent on the floor surfaces, especially dog-specific fecal bacteria. Targeted antibiotic resistance was detected from all buildings, but carbapenem resistance was only observed in the medical center. The outcome of this study can be used for further designing of future studies about quantitative dynamics of antibiotic resistance transmission and potential pathogen spread in indoor settings.

## Figures and Tables

**Table 1:** Summary of sampling locations (and areas within), floor types and traffic types.

Locations	Areas	Floor	Traffic
Medical center	Main entrance	Carpet	High
	Office	Carpet	Low
	Main entrance	Vinyl	High
	Patient room	Vinyl	Low
Veterinary hospital	Small animal office	Carpet	High
	Large farm animal clinic	Carpet	Low
	Small animal clinic	Vinyl	High
	Large farm animal clinic office	Vinyl	Low
Office/classroom building	Classroom	Carpet	High
	Office	Carpet	Low
	Laboratory room	Vinyl	Low
	Laboratory	Vinyl	Low

## Supplementary Materials

The following are available online at https://www.mdpi.com/1660-4601/16/21/4160/s1, Figure S1: Predictive margins for OTU levels from floor surfaces based on traffic levels, Figure S2: Relative abundance of bacterial community at the class level form different floor materials, traffic level, and building types, Table S1: Summary of sampling locations (and areas within), floor types and traffic types, Table S2: Bacterial richness indices of the samples presented as mean (standard deviation).
