# Supplementary material for: Indoor Microbiome and Antibiotic Resistance on Floor Surfaces: An Exploratory Study in Three Different Building Types
Source: Int J Environ Res Public Health. 2019 Oct 28;16(21):4160. doi: 10.3390/ijerph16214160 (PMC6862025; doi:10.3390/ijerph16214160)
Supplement: Supplementary file 1 [file ijerph-16-04160-s001.pdf]

Supporting Information

The Indoor Microbiome and Antibiotic Resistance on Floor Surfaces: An Exploratory Study in Three Different Building Types

Mridula Gupta[1§], Seungjun Lee[1§], Michael Bisesi[1] and Jiyoung Lee[1,2*]

[1]College of Public Health, Division of Environmental Health Sciences, The Ohio State University, Columbus, OH 43210, USA

[2]Department of Food Science and Technology, The Ohio State University, Columbus, OH 43210, USA

§ Equal contribution

*Corresponding author: Jiyoung Lee. Tel: +1 614 292 5546; Fax: +1 614 293 7710

E-mail: lee.3598@osu.edu

Table S1. Concentrations of antibiotic resistance (tetracycline, sulfonamide, and KPC) and fecal bacteria from human (HF183), ruminant (Rum2Bac), dog (BacCan), and bird (GFD) sources in floor samples from each type of building (medical center, veterinary hospital, and office). The units are gene copies/25 cm^2.

Building	Medical center				Veterinary hospital				Office building			
Floor	Carpet		Vinyl		Carpet		Vinyl		Carpet		Vinyl	
Traffic	High	Low	High	Low	High	Low	High	Low	High	Low	High	Low
Antibiotic resistance												
tetQ	7585	437	1862	339	72444	2630	34674	200	589	-	-	-
KPC	117	-	89	-	-	-	-	-	-	-	-	-
Sul1	240	234	170	85	1072	813	759	355	98	100	-	-
Microbial source tracking												
HF183	60	8	36	-	295	59	162	48	129	65	-	-
Rum2Bac	52	40	25	-	2692	1096	2291	631	-	-	-	-
Dog	398	309	269	85	7413	5012	3548	525	263	-	123	-
GFD	63	-	48	-	288	50	110	45	95	40	50	-

- Not detected.

Table S2. Bacterial richness indices of the samples presented as mean (standard deviation)

Factors		ACE	Chao1	OTUs
Floor type	Carpet	24625 (2174)	15667 (936)	6415 (217)
	Vinyl	26016 (1446)	16172 (518)	6402 (159)
Traffic type	High	25237 (1842)	16097 (511)	6548 (99)
	Low	25380 (2093)	15794 (928)	6308 (160)
Building type	Medical	25097 (2353)	15958 (904)	6387 (213)
	Vet	25603 (1569)	16117 (442)	6479 (41)
	Office	25261 (2265)	15685 (1010)	6358 (251)

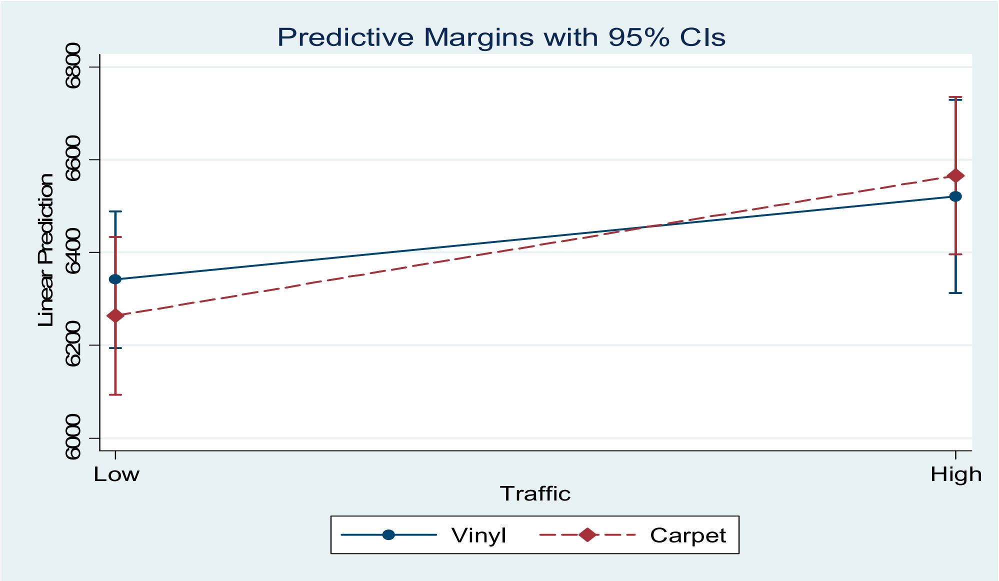

Figure S1. Predictive margins for OTU levels from floor surfaces based on traffic levels.

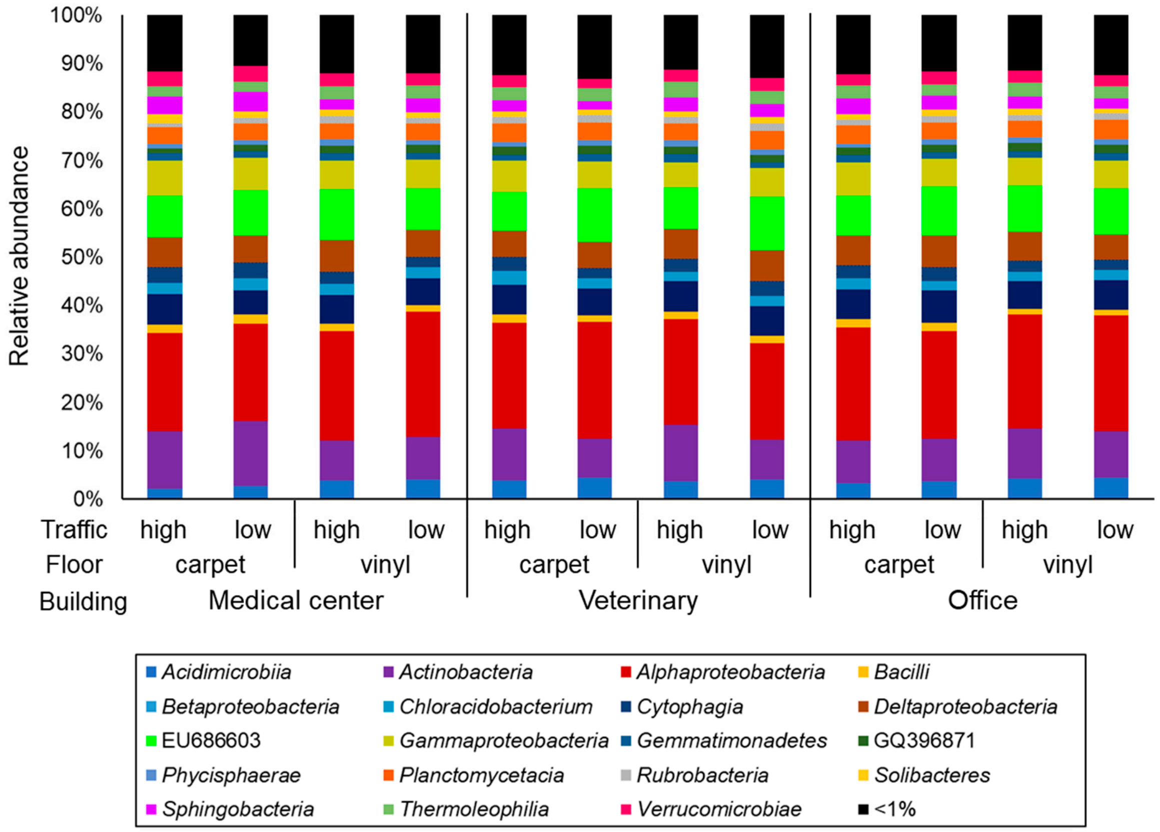

Figure S2. Relative abundance of bacterial community at the class level form different floor materials, traffic level, and building types.
